# Design and Implementation of a Multifunction Wearable Device to Monitor Sleep Physiological Signals

**DOI:** 10.3390/mi11070672

**Published:** 2020-07-10

**Authors:** Lun-De Liao, Yuhling Wang, Yung-Chung Tsao, I-Jan Wang, De-Fu Jhang, Chiung-Cheng Chuang, Sheng-Fu Chen

**Affiliations:** 1 Institute of Biomedical Engineering and Nanomedicine, National Health Research Institutes, Zhunan 35053, Taiwan; yuhlingwang@nhri.edu.tw (Y.W.); prgbruce@gmail.com (Y.-C.T.); y217834@gmail.com (D.-F.J.); cheng965@cycu.edu.tw (C.-C.C.); sanfo@nhri.edu.tw (S.-F.C.); 2 Department of Industrial Engineering and Enterprise Information, Tunghai University, Taichung City 407224, Taiwan; ijwang@thu.edu.tw; 3 Department of Biomedical Engineering, College of Engineering, Chung Yuan Christian University, Taoyuan City 32023, Taiwan

**Keywords:** Internet of Things (IoT), physiological signals, sleep physiological signals, wearable device

## Abstract

We present a wearable device built on an Adafruit Circuit Playground Express (CPE) board and integrated with a photoplethysmographic (PPG) optical sensor for heart rate monitoring and multiple embedded sensors for medical applications—in particular, sleep physiological signal monitoring. Our device is portable and lightweight. Due to the microcontroller unit (MCU)-based architecture of the proposed device, it is scalable and flexible. Thus, with the addition of different plug-and-play sensors, it can be used in many applications in different fields. The innovation introduced in this study is that with additional sensors, we can determine whether there are intermediary variables that can be modified to improve our sleep monitoring algorithm. Additionally, although the proposed device has a relatively low cost, it achieves substantially improved performance compared to the commercially available Philips ActiWatch2 wearable device, which has been approved by the Food and Drug Administration (FDA). To assess the reliability of our device, we compared physiological sleep signals recorded simultaneously from volunteers using both our device and ActiWatch2. Motion and light detection data from our device were shown to be correlated to data simultaneously collected using the ActiWatch2, with correlation coefficients of 0.78 and 0.89, respectively. For 7 days of continuous data collection, there was only one instance of a false positive, in which our device detected a sleep interval, while the ActiWatch2 did not. The most important aspect of our research is the use of an open architecture. At the hardware level, general purpose input/output (GPIO), serial peripheral interface (SPI), integrated circuit (I^2^C), and universal asynchronous receiver-transmitter (UART) standards were used. At the software level, an object-oriented programming methodology was used to develop the system. Because the use of plug-and-play sensors is associated with the risk of adverse outcomes, such as system instability, this study heavily relied on object-oriented programming. Object-oriented programming improves system stability when hardware components are replaced or upgraded, allowing us to change the original system components at a low cost. Therefore, our device is easily scalable and has low commercialization costs. The proposed wearable device can facilitate the long-term tracking of physiological signals in sleep monitoring and related research. The open architecture of our device facilitates collaboration and allows other researchers to adapt our device for use in their own research, which is the main characteristic and contribution of this study.

## 1. Introduction

The Internet of Things (IoT) involves developing technology for smart applications in fields such as healthcare [1]. An example of a healthcare application is the development of a sleep monitoring system device based on the Internet of Medical Things (IoMT) [2,3,4]. Wearable technologies for health monitoring have been maturing and expanding recently [5]. The primary use for wearable technology in a medical environment is to aid in patient or subject monitoring, which can range from simply providing a way to track the health status of patients to the remote viewing of multiple patients’ vitals [6]. Sleep status is often an influential factor related to a patient′s health status and is important to monitor [5,7]. In general, sleep is a dynamic process that varies from day to day; hence, it is important to assess multiple nights of sleep for medical, research, and wellness reasons [7,8]. Recent research has revealed that motion detection can be used to monitor sleep status [9]. Although some previous studies have indicated that motion detection alone is not enough to support precise and exact sleep monitoring, other studies have indicated that convenience and mobility are preferable over the increased precision associated with expensive and time-consuming types of medical equipment [10].

The American Association of Sleep Medicine (AASM) reported the initial practice parameters regarding the use of portable monitoring (PM) devices in the assessment of obstructive sleep apnea (OSA) in 1994 [11,12]. Many studies have been conducted to develop new methods for OSA screening in an attempt to reduce the cost and complexity of polysomnography (PSG) [13,14]. Different techniques have been proposed, with oximetry-based screening being one of the most widely suggested for both adult and pediatric populations [15,16,17]. Although these methods provide high sensitivity, they tend to have very low specificity [18]. Our device does not currently have PSG capabilities due to financial constraints. However, because we used an open architecture, we can add this capability in future iterations of the device [13].

Photoplethysmography (PPG) is an optical method that is widely used for heartbeat detection due to its low cost and convenience for detecting blood volume changes in microvascular tissue beds [19]. During heartbeat monitoring, light pulses with different wavelengths are used to noninvasively determine various physiological parameters. Typically, PPG measurement systems, such as pulse oximeters, include an optical sensor that can be easily attached to one of the patient’s appendages (e.g., finger or earlobe) [20]. This optical sensor consists of a light-emitting diode (LED) and a photodetector. The LED directs light signals into the appendage with the photodetector attached to the other side. Some portion of the light is absorbed, and the remaining light passes through the patient’s tissue. The intensity of the light passing through the tissue is measured by the photodetector. The difference between the varying levels of light absorption can be used to determine the blood volume, which can then be used to compute other physiological parameters [21,22]. However, the measured signals can be distorted by various ambient noise (optical and electrical) and by motion artifacts, resulting in measurement errors [21,23,24].

Most measurement errors caused by background noise are especially prevalent when devices are used outdoors or under direct sunlight. The manufacturers of devices that use PPG sensors employ various sampling and timing strategies to account for such noise, such as ambient light sampling. The ambient light level is measured while the deployed light transmitters are powered off and then subtracted from the signal detected when the deployed light transmitters are powered on. This approach is limited by the supply voltage level. Thus, if the measured ambient light level exceeds the supply voltage level, the sensors will overload, resulting in incorrect measurements. Measurement errors caused by motion artifacts are common when the user is moving around freely. However, motion artifacts are less of a concern for sleep monitoring applications since the user is not expected to be moving around for a majority of the time, and the simultaneous use of motion sensing can be used to exclude PPG data collected while the user moves around during sleep.

For the reasons mentioned above, we aimed to design an integrated multi-sensor device that can record human vital signs for continuous, noninvasive sleep monitoring applications. For the validation of our design concept, we implement a battery-powered, miniature, portable, and multipurpose device that can achieve a similar effect to the PSG system for sleep monitoring. Our device mainly utilizes motion detection to determine sleep duration. It includes a microphone, ambient light sensor, and PPG, which is used for heart rate (HR) measurement, allowing us to accurately assess sleep quality. Our device has the advantages of mobility and scalability compared to the PSG system [13,25]. Our aim is to allow users to be able to customize the device to their own needs by easily adding sensor components from different vendors. For this purpose, we use a plug-and-play (PnP) PPG module that can be easily modified to meet different system requirements. The resulting integrated and portable device can collect motion sensor data, and a MATLAB-based program is used to transform the data and perform reasonable and reliable sleep monitoring analyses. The motion sensor in our device is similar to that in an IMU device but directly outputs motion data. The benefit is that additional CPU power is not needed to perform these calculations.

The most important aspect of our research is the use of an open architecture. At the hardware level, general purpose input/output (GPIO), serial peripheral interface (SPI), integrated circuit (I^2^C), and universal asynchronous receiver-transmitter (UART) standards were used. With this open architecture, we can easily incorporate additional sensors, allowing us to determine whether there are more intermediary variables that can be modified to improve our sleep monitoring algorithm with multiple sensors than with only an IMU. Being able to incorporate additional sensors improves the interoperability and flexibility of our device and allows for customization according to different monitoring needs, which will improve market penetration should our device be commercialized.

At the software level, an object-oriented programming methodology was used to develop the system. Because plug-and-play sensors are associated with the risk of adverse outcomes, such as system instability, this study heavily relied on object-oriented programming. Object-oriented programming improves system stability when hardware components are replaced or upgraded, allowing us to change the original system components at a low cost. Therefore, our device is easily scalable and has low commercialization costs. The proposed wearable device can facilitate the long-term tracking of physiological signals in sleep monitoring and related research. The open architecture of our device facilitates collaboration and allows other researchers to adapt the device for use in their own research, which is the main characteristic and contribution of this research.

## 2. Materials and Methods

### 2.1. System Architecture

This research proposes that a multi-sensor device based on the IoMT infrastructure can be implemented as a wearable device comparable to the PSG system. Most comparable PSG devices merely monitor body motion by measuring variables such as motionlessness, vibration, rotation, and translation [25,26]. These measurements are then used to provide information regarding sleep status. In fact, the body is not completely motionless during sleep, and there are many undetectable behaviors that differ between sleeping and awake states [27]. According to the approaches mentioned above, data under ambient conditions will be collected using multiple sensors and factored into algorithms to aid in differentiating between sleeping and awake states with the proposed device [25].

Our device employs an Arduino Yun (Arduino YÚN, Genuino, Somerville, MA, USA). The Arduino Yun is embedded with dual microprocessors, including a 16 MHz microprocessor (ATmega32U4, Microchip Technology Inc., Chandler, AZ, USA) as a general-purpose input/output (GPIO) controller and a 400 MHz microprocessor (Atheros AR9331, Atheros Communications Inc., San Jose, CA, USA) without interlocked pipelined stages (MIPS) that has Linino OS preinstalled as a WiFi bridge. The ATmega32U4 microprocessor has 32 kB of memory, of which 4 kB is used for the bootloader, 2.5 kB is static random-access memory (SRAM), and 1 kB is electrically erasable programmable read-only memory (EEPROM). The AR9331 microprocessor uses a MIPS architecture and has 64 MB of double data rate 2 synchronous dynamic random-access memory (DDR2 SDRAM) and 16 MB of flash memory [28]. This dual architecture allows the Arduino Yun to connect to the Internet using 802.11b/g/n 2.4 GHz Wi-Fi via 802.3 protocol or 10/100 Mbit/s bandwidth Ethernet with an RJ-45 cable [28].

**Figure 1:**
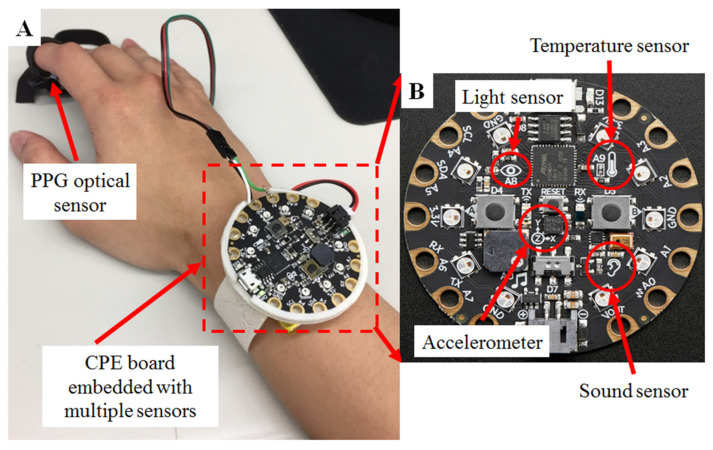
Diagram of our physiological sleep signal monitoring system, including the hardware of the portable biosignal acquisition unit. The device used a sound sensor, light sensor, triple-axis accelerometer, and temperature sensor, which were all integrated in a Circuit Playground Express (CPE) board to detect motion, ambient light, noise, and temperature. The CPE board has a diameter of 50 mm and a thickness of 1.6 mm. The collected data were stored in the 2-MB flash storage of the CPE, and the device can store up to 12 months of data from all the sensors required for data analysis. A photoplethysmographic (PPG) optical sensor (green light, 570 nm) was also included for heartbeat detection. (**A**) The system as worn on a user’s wrist with PPG sensor worn on the finger. (**B**) Close up view of the CPE board and the various sensors.

Initially, we used multiple sensor modules attached to the Arduino Yun, but the resulting device was too complex and heavy, with issues such as high-power consumption, a heavy weight, a large size, and discomfort to the wearer [28]. As a result, we switched to using an all-in-one evaluation board (EVB), such as a Circuit Playground Express (CPE) board (Adafruit Circuit Playground Express, Adafruit Industries, New York, NY, USA) [28], as shown in Figure 1A,B, as the main component [28]. The CPE is a low-cost (under USD $24) but relatively high-performance (32 bits, 48 MHz) integrated circuit board, and it includes 2 MB of flash storage for execution codes or data, 10 mini NeoPixels, a motion sensor (LIS3DH, a triple-axis accelerometer with tap detection and free-fall detection), a temperature sensor (thermistor), a light sensor (phototransistor), a sound sensor (microelectromechanical systems (MEMS) microphone), a mini speaker with an amplifier (7.5 mm magnetic speaker/buzzer), push buttons, an infrared radiation (IR) sender/receiver, an integrated circuit (I^2^C), a universal asynchronous receiver/transmitter (UART), and 8 pins that can receive analog inputs and send multiple pulse width modulation (PWM) outputs, as shown in Figure 1B. The add-on PPG sensor shown in Figure 1A was manufactured by DFRobot [28] and was used to monitor HR [28,29].

### 2.2. A Tiny Microcontroller Integrated with Multiple Sensors

CPE is a commercial product with good stability and diverse applications. Its tiny size is attractive to many developers for use in microcontroller applications. In general, most IoMT projects require a motion sensor, temperature sensor, light sensor, sound sensor, mini speaker, and push buttons for their fundamental functions. EVBs that lack the abovementioned sensors will require the addition of many more sensors, leading to problems such as I/O shortages, lengthy circuits, increased power consumption, and increased size. Additionally, lengthy code will be necessary to integrate multiple heterogeneous sensors, forcing developers to spend more time on debugging and testing.

The CPE uses an advanced reduced instruction set computer (RISC) machine (ARM) Cortex M0 microcontroller (ATSAMD21G18, Microchip Technology Inc., Chandler, AZ, USA), running at 3.3 V and 48 MHz with 2 MB of flash storage, as shown in Figure 1B. Compared to other compatible Arduino-based microcontroller units (MCUs), the CPE is more powerful and suitable for dealing with real-time data collection from multiple sensors. The sampling rate of the PPG is 200 times/second, and the 48 MHz computing power of the CPE is enough to handle concurrent PPG signal processing [25,28].

### 2.3. Motion Detection

There are many methods used to collect data for sleep physiological signal monitoring, including electrooculography (EOG) and electrocardiogram (ECG) methods, which need to be deployed in complex medical environments [5,25,26,28,30]. Because these methods are relatively expensive and time consuming for patients, an increasing amount of research is focused on the possibility of using motion detectors, such as triple-axis accelerometers, to replace electrical sensing technology [3,25,30]. Our proposed device uses the CPE’s built-in triple-axis accelerometer (LIS3DH, a triple-axis accelerometer with tap detection and free-fall detection) to collect motion sensing data, which is then translated into sleep monitoring data.

**Figure 2:**
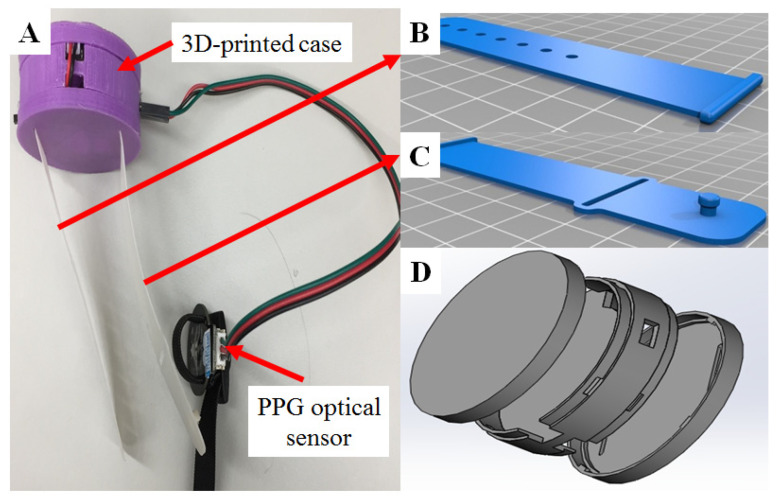
The components and an assembled schematic diagram of our wearable device. (**A**) A case with wrist straps was designed to enclose the CPE board so that the device could be worn like a watch for sleep analysis. The 3D-printed case had a diameter of 58 mm and a thickness of 65 mm. (**B,C**) Schematic of the wrist straps that were 92 mm long and 65 mm thick. (**D**) Schematic of the case enclosing the CPE board.

As shown in Figure 2A–D, we designed a shell for the CPE and then used a commercial 3D printer (K-3030, Kingtec Ltd. Hsinchu City, Taiwan) to print the components [25]. The components were integrated with the CPE and PPG modules to form our device, which could then be mounted on the user’s wrist as a wearable device (as shown in Figure 2B,C) to detect motion. The data were then analyzed and transformed into sleep monitoring data [25].

### 2.4. Heartbeat Monitor

During sleep monitoring using motion detection, deep sleep is defined as no detection of movement. However, misjudgments can occur when relying upon a lack of detection. Many studies have proposed the use of a heartbeat monitor to aid in discriminating between deep sleep and regular sleep [25,31]. Although most sleep physiological signal monitoring focuses on EOG and ECG analysis, some researchers have found that PPG is a simple and low-cost optical method for detecting changes in the blood volume of the tissue microvasculature [6,22]. Changes in blood volume occur with each heartbeat as arterial pulsations fill the capillary bed with blood. These changes in the tissue blood volume can be measured as changes in light absorbance (transmittance). The transmitted light intensity (or PPG waveform) can be plotted against time to form a small pulsatile AC waveform that can be attributed to synchronous cardiac changes in the blood volume and a large quasi-DC component that relates to the average blood volume. The DC component can slowly vary due to respiration, other sympathetic nervous system activities, and thermoregulation. Both of these optical signals can be used to determine the patient HR and other parameters, such as blood oxygen saturation (SpO_2_) and the rate of respiration [6,22].

We used a DFRobot heart rate sensor (SON1303, DFRobot, Shanghai, China) from the DFRobot Gravity Series, which uses a green LED with a 570-nm wavelength. The sensor includes built-in noise filters and issues an alarm when the HR is abnormal. According to the manufacturer’s specifications, the HR sensor has an accuracy of 98.5%. The working voltage of the HR sensor is 2.1 V, but it can withstand a maximum of 5.5 V and temperatures of −40~85 °C. The chip used in the sensor is able to achieve a bandwidth of 1 MHz at a low current consumption of 60 μA and has low input bias currents of 10 pA. Thus, our device consumes less power (operating current < 10 mA) than other devices that incorporate multiple sensors using a development board. The component cost also remained low (under USD $5 for each component).

The DFRobot HR sensor was developed based on PPG techniques and has many advantages, such as a small size (28 × 24 mm), plug-and-play connectivity, and easy integration with other wearable devices [4,26]. PPG techniques are simple, and low-cost optical methods that can be used to detect blood volume changes in the micro-vascular bed of tissues. These changes in blood flow are equivalent to the pulsatile component of the cardiac cycle. As shown in Figure 1A, the sensor can be clipped to the user’s finger or earlobe for heart rate measurement. The DFRobot HR sensor can be switched between two types of signal output modes: analog pulse mode and digital square wave mode. Therefore, the DFRobot HR sensor can be used with different MCUs and can utilize different communication modes [6,22].

### 2.5. Environment Detection

Many studies have shown that environmental light and sound have critical influences on sleep quality [23,32]. Therefore, we added ambient light (phototransistor) and sound (MEMS microphone) sensors to the proposed device for analysis, along with the motion sensor data. When motion detection using a triple-axis accelerometer is used as the sole indicator of sleep status, misjudgments can occur. We proposed the use of ambient light and sound data during sleep monitoring to eliminate any instances of misjudgment. This approach can improve the accuracy and usability of the sensor data and improve the experimental results [33].

When we collected ambient sound data, we found that the quantity of data collected was large and that sound analysis was too complex for the embedded system to handle. Thus, ambient sound analysis will be the focus of future studies. In comparison, collecting ambient light data is much simpler. Ambient lighting is generally stable, and changes in light strength are usually linear and low frequency, eliminating the need for a high sampling rate. Thus, we used ambient light data to eliminate any instances of misjudgment during sleep monitoring.

## 3. Results and Discussion

### 3.1. Core Function

This study implemented motion sensing by using the built-in triple-axis accelerometer of the CPE (Figure 1B). After user testing, feedback suggested adding a shell to enclose the CPE (without covering the light sensor). The shell was drawn and printed with a 3D printer (K-3030, Kingtec Ltd., Hsinchu, Taiwan), as shown in Figure 2B,C. The shell increased comfort for the user and also protected the CPE from any impact caused by the user moving their hand during sleep.

**Figure 3:**
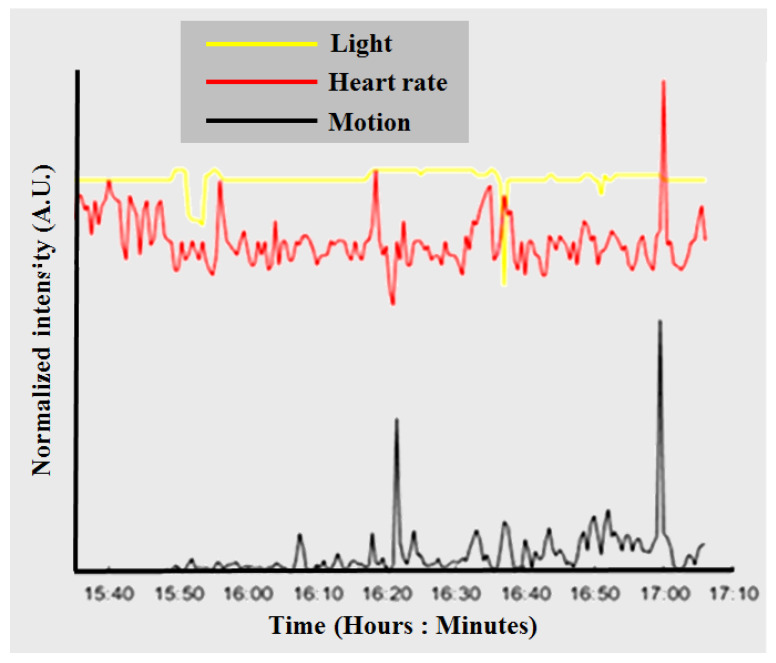
Sensor data collected from a subject wearing the developed device. The data from the light sensor are shown in yellow, the motion data are indicated in black, and the heart rate (HR) data from the PPG optical sensor are shown in red.

To calibrate and assess sleep status based on heartbeat sensing, we used an optical PPG sensor, as shown in Figure 1A and Figure 2A, which can be affixed to the human finger. The analog electrical signal from the optical sensor is processed in an electronic unit located on the CPE. For advanced analyses, the data were analyzed with the output data of the environmental light sensor as a moderator, as shown in Figure 3. The data processing and analysis were performed using MATLAB^®^ (R2007a, MathWorks Inc., Natick, MA, USA), and the results were visualized as chart-style reports [34]. The addition of the ambient light data allowed us to accurately determine sleep periods from the motion detection data. Although we were able to measure HR, we are still in the process of determining how to incorporate the data into assessing sleep quality [5,6,22,27].

### 3.2. Calibration

**Figure 4:**
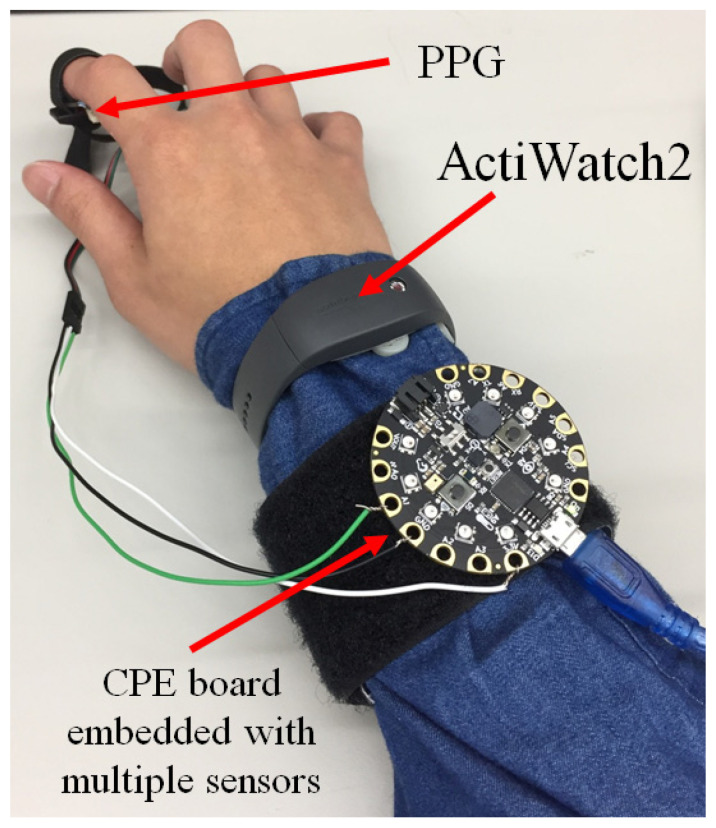
A schematic diagram for an experiment comparing our device to ActiWatch2. The subject wore both our device and ActiWatch2 on the right arm for the same time period every day for a week to collect data from both devices.

To assess the performance of our device, we compared it with a similar device currently on the market. Due to the large number of products found while surveying the market for wearable devices, we decided to limit the search to devices that were approved by the Food and Drug Administration (FDA) and were feasible for use in clinical sleep research [33,35]. We chose ActiWatch2 (Philips North America Corporation, Cambridge, MA, USA, device shown in Figure 4), which is currently approved by the FDA and has a size and functions that are suitable for use in sleep applications [33,36]. According to hardware specifications, the power of ActiWatch2 can last approximately 30 days while working at 1-min epoch lengths and storing all sensor data, vastly exceeding the requirements for our experiments [33,35].

**Figure 5:**
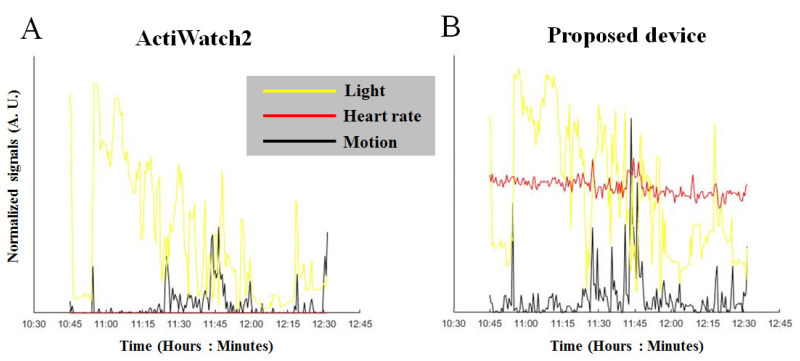
Comparison of the data collected using our device and the ActiWatch2. The data collected from ActiWatch2 are shown in (**A**), and the data collected from our device are shown in (**B**). The ambient light data (shown in yellow) collected from both devices are similar. Peaks occur at similar time points, although the slopes differ. This discrepancy could have been caused by the differences in the photoresistors used and in the locations of the light sensors on the devices. The motion sensor data (shown in black) are also similar between the two devices. Peaks occur at similar times, although the amplitudes differ. This discrepancy could have been caused by the difference in the driving and output voltages.

Data collected using the two devices are shown in Figure 5A (ActiWatch2) and Figure 5B (our device). The motion and ambient light data collected from ActiWatch2 and our device exhibited similar peaks at similar times. However, the slopes of the ambient light data (shown in yellow) obtained by ActiWatch2 and our device differed due to the different photoresistors used and the locations of the light sensors. The motion-sensor data (shown in black) were also similar for the two devices. The peaks occurred at similar times, although the amplitudes differed. These differences could have been caused by different driving and output voltages. Overall, these data demonstrate that the proposed system can collect reliable measurements similar to those of an FDA-approved commercial product (ActiWatch2).

The average power consumption of the proposed device at full function was 32–35 mAh, as measured by a KWS-V21 USB voltage and current monitoring dongle (Siqma Robotics Limited, Guangzhou, China). From user testing with the device powered by a 150 mAh, 3.7 V lithium battery, a maximum of approximately 5 h of continuous operation was achieved. Because this operation time was not sufficient for our planned 7-day testing period, the device was modified to use a 12,000 mAh external mobile power bank instead of the original lithium battery, which allowed us to keep the device continuously powered until all data collection was completed.

**Figure 6:**
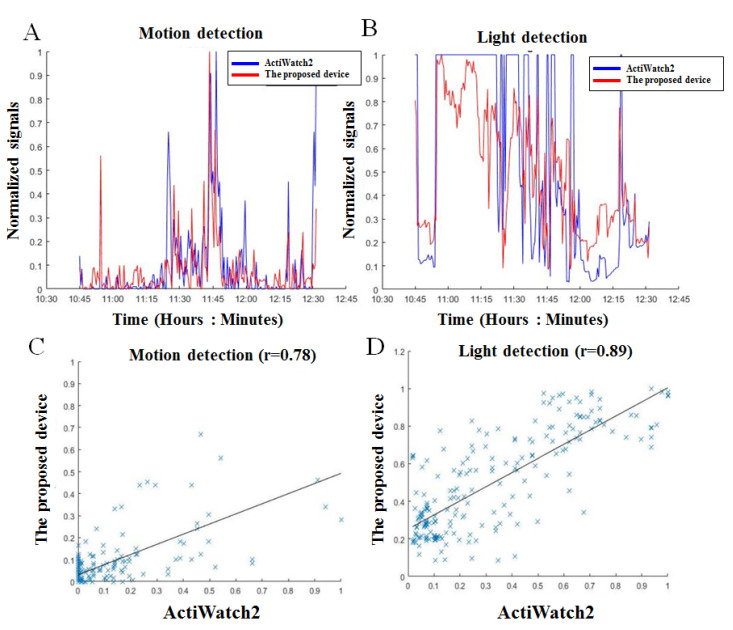
Correlation analyses of the data collected using our device and the ActiWatch2. The motion detection data are compared in (**A**). Large peaks generally occurred at the same times for both the ActiWatch2 and our device. The slight differences can be explained by the difference in the sampling frequencies of the two devices. Ambient light detection is compared in (**B**). The differences in amplitude may have been caused by differences in the photoresistors used in the two devices. The motion detection data from ActiWatch2 and our device were shown to be correlated, with a correlation coefficient (r) of 0.78, as shown in (**C**). The light detection data from ActiWatch2 and our device were shown to be highly correlated, with r = 0.89, as shown in (**D**).

After data collection for the two devices was completed, the output data were transformed into reports with the respective analysis software and output, as shown in Figure 6A,B [15,16,34]. Large peaks in motion tracking (Figure 6A) generally occurred at the same times for both ActiWatch2 and our device. Slight differences were due to the slight offset between the start time of the two devices when monitoring was turned on. The trend in the data from the light sensors was also approximately the same. Notably, the amplitudes were different, which may have been caused by the difference in the resolution of the photoresistors of the two devices, as shown in Figure 6B. The cause for the signal saturation in the light detector of the ActiWatch2 was unknown and will be investigated in further studies.

To assess the performance of the proposed device, the Pearson correlation coefficients (Figure 6C,D) were generated to compare both motion and light detection data collected from the proposed device and ActiWatch2. The motion detection data from ActiWatch2 and our device were shown to be correlated with a correlation coefficient (r) of 0.78, as shown in Figure 6C. The light detection data from ActiWatch2 and our device were shown to be highly correlated with a correlation coefficient of 0.89, as shown in Figure 6D. The equations fitted from the Pearson correlation were in the form of y=mx+b, where approximately m = 0.46 and 0.75 and b = 0.027 and 0.25 for motion and light detection, respectively [37]. Matching the sampling frequency and the photoresistor resolution of the two devices could increase these correlation coefficients [25].

### 3.3. Sleep Analysis Using MATLAB

**Figure 7:**
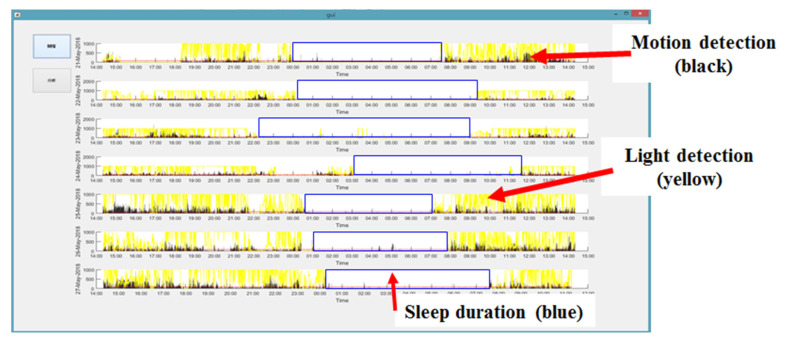
Sleep analysis was performed using MATLAB software. An example analysis of 1 week of data is shown. The data from each 24-h period are displayed separately. The motion detection data are displayed in black, the light detection data are in yellow, and the heartbeat data are in red. The blue box indicates the sleep duration, as determined from the algorithm using both motion detection and ambient light sensing data.

We developed a unified MATLAB script for user-friendly analyses of the sleep data, which were stored in the inner flash memory of the standard ActiWatch2 and our device and transferred to a desktop PC via a USB cable connection [25]. An example analysis of a week of data (from Monday to Sunday) is shown in Figure 7, in which data from each day of the week are displayed separately [25]. For the 7-day continuous testing period, the data were collected by our device at 15 bytes per data point and at a sampling rate of twice per minute. Thus, the overall size of the test data set was approximately 330 kB, which was able to be stored in 2 MB of flash storage in the proposed device. Data transmission required a USB cable connected to a PC. The data from the flash storage were transferred to the PC and displayed using the developed MATLAB sleep analysis interface. The data transmission was almost the same for ActiWatch2 as for our device [25].

The first step in the sleep analysis algorithm was to calculate the moving average of all motion data [13,25]. The second step was to clean and filter abnormal data or noises identified by discontinuities in time. The third step was to identify sleep intervals. To increase accuracy, we eliminated data from shallow sleep periods, in which sleep lasted less than 30 min, from the total sleep data set. However, multiple shallow sleep intervals were combined into one interval if the time between the intervals was short. Sleep intervals were classified as deep sleep intervals if they lasted for more than 3 h. All sleep interval data were presented to the user in chart format.

**Figure 8:**
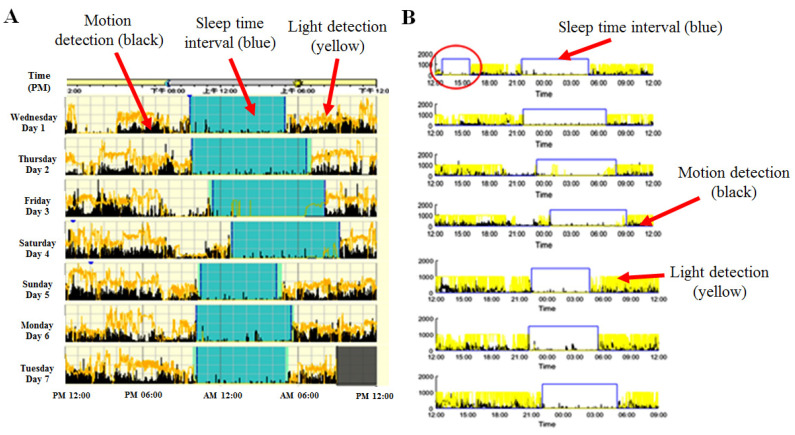
Comparison of the sleep duration analysis results using our device and using the Food and Drug Administration (FDA)-approved ActiWatch2. (**A**) The results of sleep analysis performed with ActiWatch2, with the blue sections representing the sleep duration. (**B**) The results of the sleep analysis performed based on the data collected by our device. The sleep duration is indicated by the blue line. For the experiment, each 24-h interval started at noon and ended at noon on the next day. Seven intervals were continuously recorded. The motion detection data are displayed in black, and the light detection data are displayed in yellow. The durations of sleep time recorded by ActiWatch2 from Monday to Sunday were 7.5, 9, 8.6, 8.3, 6, 7.3, and 7 h, and the durations of sleep time recorded using the proposed device were 7.7, 9, 8.1, 8.3, 6.2, 7.5, and 7.6 h. A comparison of (**A**) and (**B**) shows that there is strong agreement between the sleep analysis results from the two devices, with the exception of the interval circled in red, for which there was a false positive. This misjudgment may have been caused by the user adjusting the watch due to unfamiliarity with wearing it for the first time.

The sleep analysis results using our device were compared to the sleep analysis results using the FDA-approved ActiWatch2, as shown in Figure 8. Figure 8A shows the results of the sleep analysis performed with ActiWatch2, with the blue section representing the sleep duration for a week (from Monday to Sunday). Figure 8B shows the results of the sleep analysis for data collected from our device over the same time period. For the experimental results shown in Figure 8, each 24-h interval started at noon and ended at noon of the next day. Seven intervals were continuously recorded. The sleep duration is shown by the blue line. The motion detection data are displayed in black, and the light detection data are in yellow. The amount of sleep recorded by ActiWatch2 from Monday to Sunday was 7.5, 9, 8.6, 8.3, 6, 7.3, and 7 h, and the amount of sleep time recorded using the proposed device was 7.7, 9, 8.1, 8.3, 6.2, 7.5, and 7.6 h. A comparison of the sleep times obtained by the two devices during the week of data collection shows a significant correlation, with an exception indicated by the red circle in Figure 8B. The proposed device misjudged the 175-min interval from 13:00 to 15:55 on Monday as a sleep event. Such misjudgments are most likely caused by a first-time user being unfamiliar with wearing the sleep watch, creating a false positive [6,15,16]. This study shows that our prototype device achieved a similar level of measurement accuracy as the FDA-approved Philips ActiWatch2 for sleep applications. Our device combines motion detection, ambient light sensing, and heartbeat data for sleep analysis using MATLAB software. As shown in Figure 6, the data from both motion and ambient light sensing using our device achieved a good correlation with the measurements obtained from ActiWatch2.

For the results shown in Figure 8, each 24-h interval started at noon and ended at noon of the next day. Seven intervals were continuously recorded. The amount of sleep time recorded from ActiWatch2 from Monday to Sunday was 7.5, 9, 8.6, 8.3, 6, 7.3, and 7 h, and the amount of sleep time recorded using the proposed device was 7.7, 9, 8.1, 8.3, 6.2, 7.5, and 7.6 h. In summary, the maximum difference between ActiWatch2 and the proposed device was 7.49%, and there was no significant minimum difference. The average difference was 1.36% and the median difference was 2%. Statistically, the comparisons showed close agreement between the sleep analysis algorithms of the proposed device and ActiWatch2.

The only false positive in the sleep analysis experiment occurred during the 175 min between 13:00 and 15:55 on Monday, as indicated by the red circle in Figure 8B. This misjudgment was most likely caused by a first-time user being unfamiliar with wearing the sleep watch. Another reason may be the difference in sensitivity between the motion sensors of the two devices. Because ActiWatch2 is an FDA-approved commercial product, it has undergone rigorous and comprehensive testing. Feedback data from testing on a large number of users allowed Philips (i.e., ActiWatch2) to correct such problems more extensively than has been possible for our device [14].

## 4. Conclusions

A wearable device that employs a PPG optical sensor and multiple sensors integrated with a CPE was developed in this study. Our device enhances the performance of the PPG sensor compared to conventional apparatuses and methods. The presented sensors and methodology were integrated into a prototype device for the noninvasive, continuous, wearable, and mobile monitoring of human vital signs, such as HR, during sleep. This prototype device allows the user to read/store, process, and transmit all measurements in a single setting. Furthermore, we demonstrated the feasibility of the developed device by monitoring physiological sleep signals for 7 days. The average difference between the commercial wearable device Philips ActiWatch2 and the proposed device was 1.36%, which was not statistically significant. These results show that our proposed device is reliable and can be applied in practice. Although our device mainly relies on motion sensing to measure sleep duration, the inclusion of a PPG, microphone, and light sensor enable algorithms to be implemented in the future to more accurately assess sleep quality. In other words, our system offers comfort and convenience for users during sleeping and may therefore record information that accurately reflects user sleeping behavior. Recently, many insurance companies are seeing opportunities to incorporate data obtained from wearable devices into their risk assessments. For widespread use of wearable devices among the population, a simple device that is low in cost to produce but still has the basic functionalities of motion sensing and ambient environment assessment is necessary. Thus, the goal of our study was to produce a low-cost sleep monitoring device that can be expanded with additional sensors and is comparable to wearable devices approved by the FDA. Our future research goals are to create an integrated smart health home based on the IoMT for both healthy elderly people and those with chronic sleep issues [17,38].

Our next step will be to decrease the device size and to optimize power consumption. Although the CPE board in our device has an outer diameter of approximately 50.6 mm and a thickness of approximately 1.6 mm, all sensors and corresponding circuits can be further redesigned into an integrated chip with a smaller size (almost a 35 mm decrease in diameter) to reduce space by 30–40% after removing unused sensors and components. In other words, the significant advantages of the proposed device are its scalability and flexibility to extend its application to noise measurements, color/temperature cross-analysis, and even falling detection and position detection with the addition of sensors.

In addition, CPE is an ARM-based technology that can easily be commercialized into a powerful healthcare product. The MCU-based architecture can be applied to other convenient EVBs, such as Arduino and ARM. The next step will be to utilize big data and artificial intelligence (AI) for sleep analysis [25,39] from a large number of users wearing our device and to identify more specific factors that influence sleep [40]. For example, gaining insight into the factors that influence irregular sleep-wake rhythms can allow attending physicians to offer systematic suggestions for their users [41]. Using this approach, smart and intelligent sleeping models may be discovered and applied to smart healthcare to improve sleep quality [3,16].
